# Recommendations to improve physical activity among teenagers- A qualitative study with ethnic minority and European teenagers

**DOI:** 10.1186/1471-2458-11-412

**Published:** 2011-05-31

**Authors:** Sinead Brophy, Annie Crowley, Rupal Mistry, Rebecca Hill, Sopna Choudhury, Non E Thomas, Frances Rapport

**Affiliations:** 1 College of Medicine, Swansea University, Wales, SA2 8PP, UK; 2 Africa Educational Trust, London, WC1 6JF, UK; 3 Comic Relief, London, SE1 7TP, UK; 4 School of Health and Population Science, Birmingham University, Birmingham, UK; 5 Centre for Children and Young People's Health and Well-Being. Swansea University, Wales, SA2 8PP, UK

**Keywords:** Physical activity, teenagers, ethnic minority, qualitative, focus groups

## Abstract

**Background:**

To understand the key challenges and explore recommendations from teenagers to promote physical activity with a focus on ethnic minority children.

**Methods:**

Focus groups with teenagers aged 16-18 of Bangladeshi, Somali or Welsh descent attending a participating school in South Wales, UK. There were seventy four participants (18 Somali, 24 Bangladeshi and 32 Welsh children) divided into 12 focus groups.

**Results:**

The boys were more positive about the benefits of exercise than the girls and felt there were not enough facilities or enough opportunity for unsupervised activity. The girls felt there was a lack of support to exercise from their family. All the children felt that attitudes to activity for teenagers needed to change, so that there was more family and community support for girls to be active and for boys to have freedom to do activities they wanted without formal supervision. It was felt that older children from all ethnic backgrounds should be involved more in delivering activities and schools needs to provide more frequent and a wider range of activities.

**Conclusions:**

This study takes a child-focused approach to explore how interventions should be designed to promote physical activity in youth. Interventions need to improve access to facilities but also counteract attitudes that teenagers should be studying or working and not 'hanging about' playing with friends. Thus, the value of activity for teenagers needs to be promoted not just among the teenagers but with their teachers, parents and members of the community.

## Background

The benefits of physical activity for reducing obesity and related chronic diseases are well known [1]. Moreover, regular physical activity can provide substantial benefits to physical and mental health [2]. Individuals from ethnic minority groups are at higher risk of cardiovascular disease and diabetes [3]. Many of the risk factors associated with these conditions begin in childhood [4-6]. Evidence shows that it is during the teenage years, that girls and ethnic minority populations are most likely to develop a sedentary lifestyle, furthermore ethnicity is associated with childhood obesity [7-10]. Ethnic minority populations are less likely to participate in sport [11]. However, research targeting physical activity continues to focus primarily on white and adult populations, with less known about factors associated with physical activity habits in ethnic minority and younger populations. For example, among South Asians born and living in the UK, cardiovascular risk increases with length of residence [12-14] this suggests traditional diet and higher physical activity habits are replaced with a Westernised diet and lower activity levels when in the UK. However, physical activity is related to a set of complex behaviours in which different determinants may be relevant for different groups at different stages in life. This study examines the views of teenagers on physical activity, including what interventions the teenagers themselves would recommend to improve activity for others, with a specific focus on Somali, Bangladeshi and Welsh pupils aged 16-18. Focus group methods were used in order to explore ideas for interventions and obtain feedback to new ideas within the group. The study aims to inform the development of future complex interventions targeted at improving activity levels in teenagers.

## Methods

### Interview guide

The topic guides were developed by a multidisciplinary team including researchers, public health practitioners, teachers, parents of an ethnic minority background, and researchers from the charity Africa Educational Trust. The guide was used to ensure key themes were covered. However, free discussion of experiences and ideas were encouraged. The researchers pre-tested the interview guide with two Somali student groups; accordingly, the guide was reworded and reorganised based on feedback from the respondents. The interviews covered diet, physical activity, knowledge and recommendations for improvement of health behaviours. However, only the areas related to physical activity are presented in this paper.

### Focus group setting

This focus group study formed part of a whole school survey [15] where blood sampling (e.g. cholesterol, fasting glucose), diet diaries, aerobic fitness (20 metre shuttle test) and anthropometric measures (height/weight, skin-fold thickness, waist/hip circumference) were measured in children aged 11-13 in 10 school. In three of the schools, students aged 16-18 were invited to participate in a focus group discussion on health. Interviews were conducted and analysed by the same three researchers (AC, RM, and SB) during the school year of 2009/10. The interviews were conducted in English and all participants were required to give informed signed consent forms. The schools were selected for having a high number of ethnic minority children. However, these criteria meant that the schools were also situated in deprived areas. For two of the Somali groups (one male, one female) a community worker invited participants from various local schools to participate. For the Somali groups this additional method of recruitment was taken as participation rates of Somali pupils recruited through schools was low. Interviews lasted approximately 1 hour, were conducted during school time and were conducted in the school. In addition for the Somali groups two focus groups were held in the local leisure centre after school as this made it easier for the participants from different schools to attend.

### Participants

Participants were eligible for inclusion if they were 16+ years, considered themselves as Bangladeshi, Somali or Welsh, and were attending school. Teachers were asked to invite all eligible students to participate and those interested completed a 'consent to be contacted' form. Thus, volunteers were selected and focus group interviews were organised by word of mouth by participating children and by mobile texting with the consenting participants. There were 12 focus groups comprising of four Bangladeshi groups (2 boys, two girls groups), four Somali groups (two boys, two girls groups), four Welsh groups (two boys, two girls). The final sample size was 74 children with an average of five participants per group.

### Methods of Analysis

All focus group discussions were audio taped with the participants' consent and transcribed verbatim. A joint code book was developed from the first three interviews by the researchers (RM, AC, and SB) and additional themes were added with consensus agreement from each researcher. Each line of the transcript response was individually coded and the content analysed.

The data is presented in terms of current activity habits and recommendations to improve physical activity in the future. The groups were interviewed and the transcripts analysed, separated by gender and ethnic grouping. However, findings are presented to allow for comparison between the ethnic and gender subgroups.

Ethical review and approval was granted by the Joint Scientific Committee of the ABM University Health Board.

## Results

Response categories coalesced under several themes related to the topics of discussion. There were 32 white (17 male 15 female, 30 born in the UK), 18 Somali (11 Male 7 Female, 5 born in the UK) and 24 Bangladeshi (10 male and 14 female, 18 born in the UK) participants. The average age was 17 (range: 16-18).

### Current activity

For the girls, the main sources of physical activity were from routine tasks, especially walking locally. The Bangladeshi girls spoke of making a determined effort to walk as opposed to travel by bus; and both the Bangladeshi and Somali girls talked about gaining exercise benefits from housework as well as participating in exercises such as sit-ups and dancing in their bedroom. Somali girls reported playing games such as football with a sibling or tennis over the fence with a neighbour. Current participation in organised activities such as; acrobatics classes, swimming clubs or attending a paid gym was only discussed by the Welsh girls. Although the Bangladeshi and Somali participants spoke of their intentions to attend a gym or purchase a stationary bike, in reality, they did not go to a gym or use an exercise bike.

*Bought an exercise machine and never go on, coz I just think its not going to make any difference, I don't know why I think that I just think in the long run anyway.* (Bangladeshi girl)

Similar to the girls, the boys reported walking as a source of activity, but they also engaged in a variety of outdoor activities such as playing football or basketball with friends. The boys were more likely to use a local gym, often paying fees from their own money, rather than using the facilities provided by school. Overall, the boys were more active than the girls, with some Welsh boys helping with coaching outside school and supporting younger pupils during physical education lessons. Somali boys attended the local leisure centre (especially in winter) and reported participating in sport and daily activity.

*As long I'm doing football and basketball I'm good.* (Somali boy)

#### Barriers and motivators to exercise

All the boys talked about the positive aspects of exercise, which is something the girls hardly mentioned. For the girls, particularly the Bangladeshi girls, the only positive aspect of exercising was aiding weight loss. The boys felt that exercise was good to burn off fatty food to overcome having a bad diet. They spoke of the health benefits of exercise, and wanting to feel fit and look good. That exercise builds up muscles and gave them confidence making them feel better. The boys discussed their siblings and friends as motivators, and frequently went down to the local park to 'mess about'.

"*we have like all fatty stuff yeah and then we think "ah let's just do a bit of exercise, we'll get rid of it*" (Bangladeshi boy)

*Sports makes you look fit*. (Somali boy)

*It was actually quite good though, coz you feel proud of yourself after you've ran all that, like you know ran a long distance... and you're like you know, I've done something.* (Bangladeshi boy)

The girls however, talked about a lack of motivation, in particular, that it was difficult to start exercising; all the girls interviewed talked about a lack of time. The Bangladeshi girls felt that exercise could have a negative effect on school work and revision in terms of making a person more tired and making concentration harder. The Somali girls felt that if a person was not fat there was little need to exercise, that only overweight people should exercise. For the Welsh girls, access to facilities outside school was too complicated, for example, the cost of joining a club was expensive and they did not have access to the facilities at school. However, in school it was the hassle of feeling sweaty after activity and no proper showers that were the barriers to exercising.

**That's what my family says, you skinny why are you exercising, you're skinny, you don't need to exercise. **(Somali girl)

(Regarding access to swimming) *But you've got to make sure that there isn't anything* (clubs) *there and sometimes you have to like book a lane before, you can't just go in and go swimming* (Welsh girl)

The Bangladeshi and Somali girls spoke of the embarrassment factor of exercising and of being too shy to do sports with others. They felt there was no encouragement to exercise from their family. This led to the sense of being de-motivated and not being bothered to exercise. The girls in one Bangladeshi group spoke of a community club that their mothers had joined. Their mothers had begun exercising through the club, taking exercise classes and walking. However, the girls had little interest or information about the club and what their mothers were doing.

*I think it's easier for boys because they can go out more and they can go up to the park and play football and they won't get told off by their parents because they're looking at you. *(Bangladeshi girl)

The girls were quite passive about finding out about what activities were available. Somali girls said they did not know what was available, both in and out of school, as there was no advertising or publicity. They knew of 'girls only' teams when they were younger, but didn't know if these continued to exist for older girls. They felt that this could be because there were not enough older girls interested to make a girls team.

*I used to bring my kit in almost every day but like people weren't doing it so then I got bored because there wasn't enough people doing it with me so I was like "What the hell is the point"* (Somali girl).

For boys, the main barriers included poor access to facilities, and limited opportunities for participation in physical activity due to the requirement of supervision by adults and teachers. They felt access to school facilities was restricted, for example, the goal centre/playing fields and pool are closed after school and used for lessons during school time. They expressed a need to access school facilities, such as playing fields and pitches, outside of school hours and reported that during the day there has to be a teacher supervising the use the school facilities but there were rarely teachers available. The boys mentioned that afterschool activities are more readily available for the younger schoolchildren. Although they wanted more freedom to use the school sports facilities, they wanted to use places where they could to simply 'hang out' with friends and not be supervised by adults. In general, they felt health and safety restrictions (i.e. 'avoiding injury' and the 'school being sued') made it harder to be active. Although the boys recognised that they could pay to become a member of sports club or gym, this was regarded as too expensive an option.

*We don't want to like go there and a teacher telling us "do this, do that" we just want to go and you know, just do what we want like if it's a football...we just go and play football rather than doing this and that with the teachers...'Cos we organise it ourselves...*(Bangladeshi boy)

The Somali boys felt sport was now very popular but that there were not enough facilities for everyone. Even with the government funded initiatives, the Welsh boys reported that there were a limited number of coaches and not all the people who wanted to participate were able to, as it was oversubscribed. Indoor facilities such as the leisure centre were all over booked and overcrowded, and the Somali boys felt that outdoor activities were limited by the weather. Specifically, they complained that the sun sets early in winter leaving little time after school, that it rains too much and that their parents think that if they play outside they will get a cold, so they have to stay in. The Somali and Bangladeshi boys suggested their families were not very supportive of them doing exercise. This was because of the worry of injury, for example in the gym 'You might drop a weight on your foot' (Bangladeshi boy). The Welsh boys reported that their parents said 'Now you are 16 I am not giving you any more money" (Welsh boy); and so the need for a part time job coupled with their academic workload left no spare time for exercise. Many recreational and sports facilities were reported as not within walking distance, and only those with supportive parents were transported to activities. Therefore, for the boys, there was a good level of motivation but they felt frustrated at the lack of access to good facilities and too much adult supervision of their activities.

### Future recommendations for physical activity

#### Attitudes to activity

When asked "what would you do to improve physical activity for other children?", the Bangladeshi and Somali girls felt a need for greater community action in order to improve attitudes towards girls exercising. The girls wanted to be able to enjoy active pursuits with friends, stating that this would encourage them and make it easier to be active. All the participating girls in the survey felt that there were more opportunities for boys including better provision of activities as well as reporting that, the boys 'took over' available activities, and spaces that encourage physical activity, such as parks. The Somali girls wanted family activities as well as girl's only facilities.

*Doing it in groups is easier like a group of friends doing something, it makes it more fun ...'cos like religion comes into it and culture and everything, basically stuff like that. But me, I would rarely go running, I used to a long time ago, I still go out running but then it's like it's just like your parents...*(Bangladeshi girl)

*Like Islam like women can't be places where the men are so they can't exactly go play tennis with their trousers and stuff, but like the family can go because it's okay for them to see them in trousers, so stuff like that* (Somali girl)

In contrast, the Bangladeshi girls did not want to exercise with their mothers. Whilst they were aware that their mothers exercised in a woman only walking group, they did not want to be a part of it.

The Welsh girls, similar to the Somali and Bangladeshi girls, wanted to improve attitudes to activity. They suggested a number of initiatives such as; celebrity endorsement to make activity popular, 'health checks' so that individuals were aware of the consequences of an unhealthy lifestyle and understood the benefits of activity. They thought parents should be better educated as to how to give their family a healthier life. All groups felt that there should be shock tactics to push people into seeing the consequences of inactivity. They felt that the school should give more 'healthy living' type lessons, prevent them from sitting in the sixth form common room, and provide them with the facilities to be more active.

*I think they should encourage people to not use the sixth form block as well, because we're just wasting our time in there, just sit there. I'm gonna be sitting there until twelve. *(Welsh girl)

#### Supervision and type of activity

All the girls identified a need for a greater variety of activities, with less emphasis on exercise and more on 'getting together', such as dancing, tennis, basketball, and badminton on the beach, or girls football. For example, they said that events organised by the school in the summer only lasted a few weeks but were fun. The girls said it was possible to try new activities such as archery, boxing and wall climbing, and that parents were happy for the girls to participate as it was supervised and organised by the school. However, during term time they felt the problem was that most school activities needed to be supervised and that there were not enough people to do the supervising outside holiday times. The Bangladeshi girls would like a 'trainer' to motivate and help push girls to exercise during term time and during exams and also to overcome the shyness barrier. In addition they felt that getting older children more involved in delivering activities would help everyone. For these girls, the solution to the lack of teachers could come from using older children to help coach and supervise the younger children and to show them the benefits of exercising. This was something that the boys were already doing. The Somali girls thought there should be better provision of information, particularly for families.

*It's got to be organised because then everything is organised and everyone is going together and it's a lot safer and parents don't mind sending you off, like school trips and things, they don't mind you going to those because they know that you're with the school. And so if the school did a lot more of those then it'd be easier for people to go like you're saying, but your mum not being okay with it, then that would probably make it a bit more okay.* (Bangladeshi girl)

Whereas the girls wanted supervised 'safe' activities, the boys wanted fun activities without constant teacher supervision. They wanted better access to school facilities outside of school hours, youth groups and other places where you could 'chill' with friends. They felt more facilities should be provided as there was not enough to meet demand.

*... they need to have a lot more of these facilities available. It may cost more money but that's the only way I think you will get more people active, by giving them a choice.* (Welsh boy)

#### Cost of activity

The boys felt that cost was a barrier to organised activities and believed that the government should make these free of charge to users. In fact some of the Somali boys thought there should be prizes and incentives to exercise.

*The point is like every person is good at something, as I say if you are good at running, run if you are good at football, play football. If you want to get some small children like do something you just tell them you're going to win £15 if you beat all these people by doing football or running. *(Somali boy)

#### How activity is promoted

The boys thought that after school clubs should include more physical activities and that if pupils could stay after school that this would be a good opportunity to do exercise. The boys wanted healthy lifestyle information to be given by '*People you like and admire*' (Bangladeshi boy) and to use the older children to help engage the younger children.

"*Because they listen to us and not the teacher"* (Welsh boy).

*It's cooler to talk to a sixth former, so they would come to me to talk about health and fitness and all that. And I'm just like... coz I had one who was smoking and I told "you know you really don't want to be doing that" and he's taken it on board ... but I felt he had more of a connection with me than the teacher because he shouldn't do that and the teacher might tell the parents.* (Welsh boy)

The children thought the Personal and Social Education lesson should concentrate more on health, and less on sex and puberty. At the moment these lessons were thought to be 'pointless'. They thought health should be incorporated into existing lessons, for example, into science or other such lessons that are taken seriously.

*They teach us about drugs and they teach us about alcohol and they teach us about sex but they don't teach us something like healthy eating. They just scared about us like having sex and doing drugs basically.* (Welsh girl)

#### Involving parents and community

The Welsh boys suggested that the mind sets of some people needed to be changed and that this could be done through the media and by targeting parents as well as children. The boys felt upbringing was an important part of how you live your life. They remarked parents needed to be educated in how to bring up their children to be healthier.

## Discussion

This study confirms that girls in this age group, especially those from ethnic minority groups, do much less exercise than boys. It also illustrates that, in general, while girls have a fairly negative attitude towards physical activity, boys have a positive approach. All participants indicated that the family, the school, and society in general, are not supporting or encouraging them to exercise. Lack of support was put down to a variety of reasons. These included, exercise for girls not being regarded as important, fear of injury or illness for boys such as getting a cold outside, and a belief that teenagers should be spending their time studying or working, rather than playing outside. Therefore, the boys and girls in this study regardless of ethnic background, focused on interventions that changed community attitudes to activity. They suggested that there should be more education and information for parents, not just for children. Both boys and girls suggested that information about the benefits of physical activity should be presented within a serious context, for example as part of a biology lesson instead of part of an 'unexamined pointless' subject (such as Personal and Social Education). They remarked that there was a need for more facilities and that these should be aimed at what children want, for example, enabling fun activities with friends. In addition, the stereotypes regarding girls especially for ethnic minority Muslim girls, could be questioned as the girls talked about wanting the opportunity to play football, try boxing, wall climbing and have physically active fun with friends, just like the boys. These activities could be supervised for the ethnic minority girls, but not necessarily for boys. For the boys, systems were already in place that allowed older boys to help deliver activities for younger boys; this was a system that girls would like to be available for them.

### Limitations

This study interviewed teenagers aged 16-18 only who volunteered. Accordingly, this sets the children apart from those who have left school at age 16 and who were not interested in health or research. Thus, although the children came from deprived areas they are, by selection, the better educated and more outspoken children in this area. There was some difficulty in engaging the Somali girls in this study, and although 10 girls agreed to participate in each focus group (20 in total), only 3 came to the first group and 4 came to the second group. There appeared to be partly a lack of communication between the girls and the school, for example, one group were told by a teacher to return to class but did not explain to the teacher that they were coming to the focus group (this did not occur with the boys or the Bangladeshi or Welsh girls). For the second group, the girls organised the groups on a day when the school had a school trip. However, even when they were available to come, some of the girls were unwilling to attend despite having previously agreed. The girls were reluctant to talk when the tape-recorder was on but were happier to talk after the focus group was finished. For at least one girl communication in English was difficult so there was a reliance for others in the group to translate. Therefore, one to one interviews in the girls own homes, with the option of an interview in Somali and possibly with appropriate incentives, may be a more successful method of data capture for the Somali girls group.

### Findings in context

Previous ethnic specific interventions have focused on involving both family and community organisations [16] and while this is supported by the Somali girls in this study, it was not supported by the Bangladeshi girls. The Bangladeshi girls were most positive about participating in school based activities with friends (to be acceptable to parents) and none mentioned provision at the Mosques or in community specific groups. In fact, being part of ethnic minority women's groups was something for their mothers but not something they wished to join. This may be a migration factor, in that the Bangladeshi children were generally born in the UK, attend local schools, and so are integrated in a culturally mixed environment and would like to do activities with their friends who may also of different ethnic, religious and cultural backgrounds. Thus, while interventions aimed at adults in ethnic minority communities focus on walking, sports groups and community based programmes [17]; this may not be the best approach for the young people. They expressed a wish to participate in 'fun activities with friends' and that although this would be with their parents encouragement, it would not necessarily be in community groups [18]. In this study, teenagers (Somali/Bangladeshi/Welsh age 16+) identified the same needs as white overweight youths (age 14-16) [19], namely family support, physical activity encouragement and a physically active environment at school. As shown previously [13], parents were not necessarily supportive but children received their support from friends and siblings. They identified the same barriers as college youths in the USA [14], specifically facilities were over crowded and intimidating. This would suggest, interventions aimed at teenagers need to address the same generic barriers working on community and family support, regardless of age or ethnicity.

## Conclusions

This study is important because people's current attitudes to physical activity are strong determinants of their likelihood for physical activity in the future [20]. Interventions should focus on changing the attitudes of parents, communities and society toward activity. The children stressed the need to both recognise, and support, girls in being active, and to give more freedom to boys rather than focusing on the formal supervision of activity and on health and safety concerns. The findings from this study can provide the foundations and 'active ingredients' [21] to improve the design of future youth-focused physical activity interventions.

## Declaration of Competing interests

The authors declare that they have no competing interests.

## Authors' contributions

All authors were involved in the design, RM and AC and SB conducted and analysed the focus groups and drafted the initial manuscript. All authors gave comments and amendments and gave approval to the final version.

## Pre-publication history

The pre-publication history for this paper can be accessed here:

http://www.biomedcentral.com/1471-2458/11/412/prepub
